# Numerical Model to Analyze the Physicochemical Mechanisms Involved in CO_2_ Absorption by an Aqueous Ammonia Droplet

**DOI:** 10.3390/ijerph18084119

**Published:** 2021-04-13

**Authors:** M. I. Lamas Galdo, J. D. Rodriguez García, J. M. Rebollido Lorenzo

**Affiliations:** 1 Escola Politécnica Superior, Universidade da Coruña, 15403 Ferrol, Spain; 2 Escola Universitaria Politécnica, Universidade da Coruña, 15405 Ferrol, Spain; de.dios.rodriguez@udc.es; 3 IES de Valga, 36645 Xanza, Pontevedra, Spain; navalrebollido@hotmail.com

**Keywords:** carbon dioxide, CO_2_, absorption, CFD

## Abstract

CO_2_ is the main anthropogenic greenhouse gas and its reduction plays a decisive role in reducing global climate change. As a CO_2_ elimination method, the present work is based on chemical absorption using aqueous ammonia as solvent. A CFD (computational fluid dynamics) model was developed to study CO_2_ capture in a single droplet. The objective was to identify the main mechanisms responsible for CO_2_ absorption, such as diffusion, solubility, convection, chemical dissociation, and evaporation. The proposed CFD model takes into consideration the fluid motion inside and outside the droplet. It was found that diffusion prevails over convection, especially for small droplets. Chemical reactions increase the absorption by up to 472.7% in comparison with physical absorption alone, and evaporation reduces the absorption up to 41.9% for the parameters studied in the present work.

## 1. Introduction

The concentration of CO_2_ in the atmosphere is increasing at an alarming rate. This trend is causing irreversible climate change, such as atmospheric warming, more extreme weather events, rising sea levels, etc. Since the main anthropogenic source of CO_2_ emissions is the combustion of fossil fuels, reducing CO_2_ in combustion systems is a crucial task. Mainly, three CO_2_ elimination approaches have been developed in the recent years: post-combustion capture, pre-combustion capture, and oxy-combustion. Compared to the other two technologies, post-combustion capture is easier to implement in existing power plants. It is currently the most mature process for CO_2_ capture [1,2] and has also been successfully applied for SO_x_ from many years [3,4,5,6].

Among the most commonly used post-combustion methods for CO_2_ elimination are membrane separation, cryogenic separation, adsorption, physical absorption, and chemical absorption. In membrane separation, membranes allow only CO_2_ to pass through, and other flue gas components are excluded. Cryogenic separation is based on CO_2_ separation by condensation. That is, flue gas containing CO_2_ is cooled to condensation and then separated from other gases. Adsorption is a physical process that involves attaching gas or liquid to a solid surface. Physical absorption involves CO_2_ absorption into the solvent, while in chemical absorption CO_2_ reacts with a solvent. Typical solvents are amines, aminoacids, alkalonanimes (DEA, MEA, MDEA, etc.), ammonia, alkalis (NaOH, K_2_CO_3_, Na_2_CO_3_, etc.), piperazine, etc. Chemical absorption is widely employed due to higher selectivity and capture efficiencies, even at low CO_2_ concentrations [7,8,9,10,11].

Alkalis are the most representative solvents for chemical absorption. Both strong alkaline solutions, such as KOH or NaOH, and weak ones, such as alcohol amines or ammonia, are employed on a wide scale. The main disadvantages of strong alkaline solutions are the corrosion caused to the equipment and difficulties on the resourcelization of the products. Alcohol amines have also been widely used. However, a great deal of energy is needed for regeneration and may also cause corrosion to the equipment. In recent years, ammonia has been a popular alternative; it has high absorption efficiency and absorption capacity, as well as low energy requirements for absorbent regeneration. Moreover, resources are widely distributed. Another advantage is that ammonia can also reduce other pollutants, including SO_2_ and NO_x_ [12,13,14,15,16].

Chemical absorbers include, among others, bubble reactors, sieve-plate towers, packed bed scrubbers, magnetic stirred tanks, and spray towers. Of these, spray towers are the most widely employed system due to their low pressure drop, simplicity, and low maintenance costs. Several experimental works can be found in the literature about the effect of the liquid and gas flow rate, CO_2_ initial concentration, temperature, pressure, etc. Nevertheless, to have a thorough grasp of the CO_2_ absorption process, it is necessary to complement experimental measurements with numerical methods. In this regard, one can refer to Javed et al. [17] and Javed et al. [18], who investigated how cyclonic flow enhances CO_2_ absorption; or Sadripour et al. [19] and Kavoshi et al. [20], who analyzed the effect of the gas temperature, absorbent concentration, and liquid to gas flow rate ratio. The main limitation of these numerical works is that a simulation of the complete spray tower does not provide information about the interior of each droplet. Several papers about single liquid droplets can be found in the literature. One can refer to Choi et al. [21], who analyzed the chemical absorption of CO_2_ in a droplet of aqueous NH_3_ and developed a correlation to characterize the wall flux as a function of different parameters. Juncu [22] examined the influence of the diffusivity ratio on the mass transfer rate. Wylock et al. [23] studied the interactions between the diffusive and convective mass transports and chemical reactions. Chen et al. [24] compared Selexol, Rectisol, and water as solvents. Pawelski et al. [25] investigated CO_2_ absorption into a droplet of a cyclohexanol/methyl chloroacetate/sodium hydroxide solution.

This study focuses on a CFD model for analyzing CO_2_ absorption using ammonia as absorbent. Since the main objective is to characterize the governing mechanisms involved, a single droplet was analyzed. The time evolution of the concentration fields, velocity, pressure, and temperature was characterized. This provided information for analyzing the influence of the main physicochemical processes responsible for the rate of absorption, such as diffusion, solubility, convection, and chemical reactions. Additionally, the effect of droplet evaporation was analyzed too. Unlike other numerical models mentioned above, the present work takes into account in the same model both liquid and gas phases, includes the evaporation effect, characterizes the droplet diameter against time, analyzes physical and chemical absorption separately, and studies the effect of both liquid and gas temperatures.

## 2. Numerical Implementation

**Figure 1:**
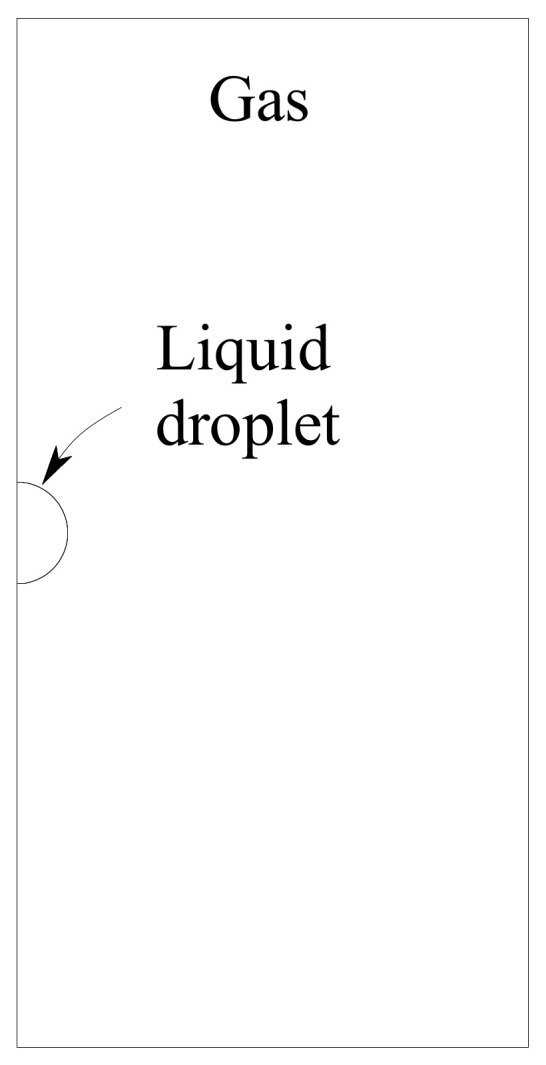
Computational domain.

The model proposed in the present paper analyzes a single droplet that falls counterflow with respect to an exhaust gas stream containing CO_2_. The computational domain is provided in Figure 1. The domain size was a 15 × 6 droplet radii from the droplet center. It was verified that these dimensions eliminate any potential effect of the outer boundaries on the flow close to the droplet. Several tests were performed to set the most appropriate dimension for the computational domain. The problem was simulated as axisymmetric.

When viewed in a fixed coordinate system, the droplet and gas move in relation to each other. Modeling this movement requires a large computation domain and, thus, high computation time. An alternative approach is to work with a coordinate system that moves with the droplet. In this case, the droplet is fixed in space and the relative droplet-gas velocity is imposed as inlet and outlet boundary conditions, i.e., the gas flow enters and leaves the computational domain at the relative velocity.

### 2.1. Chemical Model

At the liquid-gas interface, the gaseous CO_2_ dissolves in water and its concentration obeys Henry’s law, Equation (1):

(1) $$C_{CO_{2}}=p_{CO_{2}}k_{H}$$

whereby *C_CO_*_2_ is the concentration of CO_2_ in the solution, *p_CO_*_2_ the partial pressure of CO_2_, and *k_H_* the Henry’s constant, expressed as, Sander [26]:

(2) $$k_{H}=k_{H}^{0}e^{\frac{-\Delta H_{\mathrm{soln}}}{R}\left(\frac{1}{T}-\frac{1}{T^{0}}\right)}$$

whereby $k_{H}^{0}$ is the Henry’s constant at the reference state, $\Delta H_{\mathrm{soln}}$ the solution enthalpy, *R* the gas constant, *T* the absolute temperature, and *T^0^* the reference state temperature. Values of *T^0^* = 298.15 K, $k_{H}^{0}$ = 0.034 M/atm, and −$\Delta H_{\mathrm{soln}}$/*R* = 2400 K were employed [26].

Inside the droplet, the reactions (3)–(6) were assumed [27]. It is worth mentioning that, due to the low temperatures employed in the present work, there should be formation of solid ammonium hydrogen carbonate, NH_4_HCO_3_, which is a chemical species not considered in this chemical model.

(3) $$NH_{3}+CO_{2}\overset{k_{f1}}{\underset{k_{b1}}{⇄}}NH_{2}COOH$$

(4) $$NH_{2}COOH+H_{2}O\overset{k_{f2}}{\underset{k_{b2}}{⇄}}NH_{4}HCO_{3}$$

(5) $$CO_{2}+H_{2}O\overset{k_{f3}}{\underset{k_{b3}}{⇄}}H_{2}CO_{3}^{}$$

In these reactions *k_fr_* is the forward rate constant for the reaction *r* and *k_br_* the backward rate constant, shown in Table 1 [27,28].

### 2.2. Transport Equations

The level-set method was employed to difference both liquid and gas phases. The conservation of mass and momentum, Equations (6) and (7), were solved for both phases. The energy conservation equation, Equation (8), was also applied. Inside the droplet, the energy equation was solved until the evaporation temperature was reached. Once this temperature is reached it is set as constant and a droplet volume reduction due to phase change is implemented.

(6) $$\nabla \cdot \vec{u}=S_{m}$$

(7) $$\rho \frac{\partial \vec{u}}{\partial t}+\rho \nabla \cdot (\vec{u}\vec{u})=-\nabla p+\rho \vec{g}-\nabla \cdot \tau +S_{mo}$$

(8) $$\rho c\frac{\partial T}{\partial t}+\rho c\nabla \cdot (\vec{u}T)=k\nabla^{2}T+S_{e}$$

whereby *u* is the velocity, *ρ* the density, *t* the time, *p* the pressure, *g* the gravitational acceleration, *τ* the stress tensor, *c* the specific heat, and *k* the thermal conductivity. The source term *S_m_* of Equation (6) accounts for the phase change, and is given by [29]:

(9) $$S_{m}=\left(\frac{1}{\rho_{g}}-\frac{1}{\rho_{l}}\right)m$$

whereby $m=\nabla Hh(T_{e}-T_{g})/L_{v}$. The expression *h*(*T_e_ − T_g_*) represents the heat transferred between the droplet and gas, and, thus, *h*(*T_e_ − T_g_*)/*L_v_* represents the mass transfer per unit area along the interface. *T_e_* is the evaporation temperature, *T_g_* the gas temperature, *L_v_* the latent heat of evaporation, and *h* the heat transfer coefficient, and is given by [30]:

(10) hDk=2+0.6Re1/2Pr1/3

whereby *D* is the droplet diameter, *k* the thermal conductivity, *Re* the Reynolds number, and *Pr* the Prandtl number.

*H* is the Heaviside function, Equation (11), which was assumed as 0 for the liquid, 1 for the gas, and a value between 0 and 1 along the interface. As mentioned above, the interface was treated using the level set method. This procedure is based on a scalar magnitude, the level set, ϕ, which represents the distance from the interface. The interface corresponds to a zero value of the level set. In the present work, a negative sign of ϕ was adopted for the gas and positive for the liquid.

(11) $$H=\left\{\begin{array}{ll} 0 & \mathrm{if}\;\phi <\varepsilon \\ 1 & \mathrm{if}\;\phi >\varepsilon \\ \left(\phi +\varepsilon )/(2\varepsilon )+[\sin(\pi \phi /\varepsilon )]/(2\pi \right) & \mathrm{if}\;\left|\phi \right|\leq \varepsilon \end{array}\right.$$

The source term *S_mo_* included in the momentum conservation equation, Equation (7), accounts for the effect of surface tension, given by [31]:

(12) $$S_{mo}=\sigma \kappa \nabla H$$

The source term *S_e_* included in the energy conservation equation, Equation (8), accounts for the phase change, and is given by:

(13) $$S_{e}=L_{v}m$$

whereby *κ* is the curvature of the interface, given by:

(14) $$\kappa =-\nabla \cdot \hat{n}$$

whereby $\hat{n}$ is the unit vector in the normal direction of the interface, given by:

(15) $$\kappa =\frac{\nabla \phi }{\left|\nabla \phi \right|}$$

In order to take into account the movement of the interface, its propagation was characterized through the following equation:

(16) $$\frac{\partial \phi }{\partial t}+{\vec{u}}_{\mathrm{int}}\cdot \nabla \phi =0$$

whereby ${\vec{u}}_{\mathrm{int}}$ is the velocity if the interface, which can be obtained through a mass and heat transfer balance at the interface, given by:

(17) $$\vec{m}=\frac{h(T_{e}-T_{g})}{L_{v}}=\rho ({\vec{u}}_{\mathrm{int}}-\vec{u})$$

In order to preserve the characteristic of being distance from the interface, the level set function must be reinitialized at each time step. To this purpose the following equation was employed:

(18) $$\frac{\partial \phi }{\partial \xi }=sign(\phi_{0})(1-\left|\nabla \phi \right|)$$

whereby *ξ* is an artificial time introduced because Equation (18) is a temporal equation which must be solved at each time step, and $\phi_{0}$ is the level set field at the start of each iteration in this artificial time, i.e.,

(19) $$\phi (x,0)=\phi_{0}(x)$$

Regarding the chemical treatment, it was assumed that the gas is non-reacting. Nevertheless, in order to take into account species transport in the liquid droplet, the following equation was solved for each *i*th species:

(20) $$\frac{\partial C_{i}}{\partial t}+\nabla \cdot (\vec{u}C_{i})=\nabla \cdot \left(D_{i}\nabla C_{i}\right)+R_{i}$$

whereby *C_i_* is the local concentration of each species, *D_i_* the mass diffusion coefficient, and *R_i_* the net rate of production of species *i* by the chemical reactions indicated in Equations (3)–(5), given by:

(21) $$R_{NH_{3}}=k_{f1}C_{NH_{2}COOH}-k_{b1}C_{NH_{3}}C_{CO_{2}}^{}$$

(22) $$R_{CO_{2}}=k_{f1}C_{NH_{2}COOH}-k_{b1}C_{NH_{3}}C_{CO_{2}}^{}+k_{f3}C_{H_{2}CO_{3}}-k_{b3}C_{CO_{2}}^{}$$

(23) $$R_{NH_{2}COOH}=k_{f1}C_{NH_{2}COOH}-k_{b1}C_{CO_{2}}{+k}_{f2}C_{NH_{4}{HCO}_{3}}-k_{b2}C_{NH_{2}COOH}$$

(24) $$R_{NH_{4}{HCO}_{3}}=k_{f2}C_{NH_{4}{HCO}_{3}}-k_{b2}C_{NH_{2}COH}$$

(25) $$R_{H_{2}CO_{3}}=k_{f3}C_{H_{2}CO_{3}}-k_{b3}C_{CO_{2}}$$

### 2.3. Boundary Conditions

As mentioned earlier, a reference frame that moves with the droplet was employed. A free-stream velocity was imposed as inlet and outlet boundary conditions. The drag force tends to reduce the relative velocity between the droplet and gas, and gravity tends to increment it. This was implemented in the numerical model by adjusting the free-stream velocity at each time step. According to this, the acceleration or deceleration is given by:

(26) $$\frac{du_{\infty }(t)}{dt}=g-\frac{F_{drag}}{\rho_{l}\pi D^{3}/6}$$

whereby *u_∞_* is the free-stream velocity (gas velocity) and *F_drag_* the drag force, computed through the following expression:

(27) $$F_{drag}=\frac{C_{D}A_{f}\rho_{g}u_{\infty }^{2}}{2}$$

whereby *A_f_* is the reference area (for a sphere π*D*^2^/4), and *C_D_* the drag coefficient, obtained by the following expression [32]:

(28) CD=24Re1+0.15Re0.687

Finally, the variation of the free-stream velocity during a time step Δt is:

(29) $$u_{\infty }(t+\Delta t)=u_{\infty }(t)+\Delta t\frac{du_{\infty }(t)}{dt}$$

### 2.4. CFD Model

**Figure 2:**
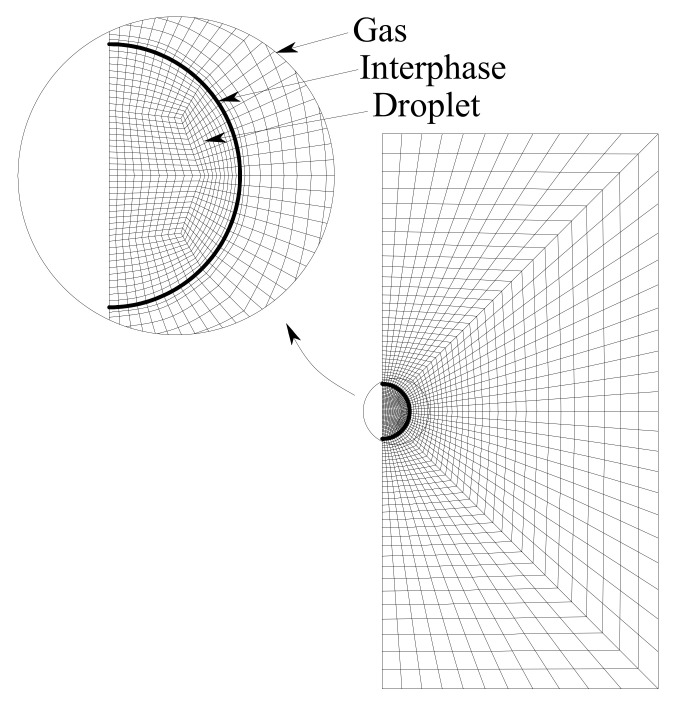
Computational mesh.

The computational mesh is indicated in Figure 2. The grid size is uniform in the tangential direction using Δθ = 4°. In the radial direction, the mesh is finer inside the droplet and near the interface. The mesh is made up of tetrahedral elements, and it was modified at the center of the droplet to avoid convergence problems.

Several meshes were tested to verify the adequacy. The model was simulated using the open software OpenFOAM (Open Field Operation and Manipulation, Reading, UK). This software was chosen because the code can be completely manipulated. A new OpenFOAM solver was programmed for the present study using the C++ programming language. OpenFOAM is based on the finite volume method. The pressure-velocity coupling was treated using the PISO (Pressure Implicit Splitting of Operators) procedure. The equations were discretized by QUICK interpolation and the temporal treatment was solved by an implicit method.

### 2.5. Validation

**Figure 3:**
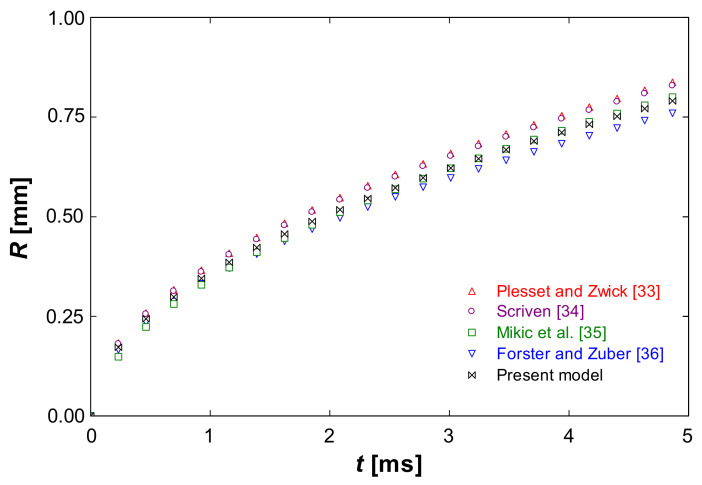
Bubble radius (R) against time (t) applied to a spherical water bubble growing in a 5 °C superheated liquid medium.

This evaporation model was validated with experimental results developed elsewhere. Several authors reported data about spherical bubble growing due to evaporation, Plesset and Zwich [33], Scriven [34], Mikic et al. [35], Forster and Zuber [36], etc. These authors developed correlations based on their experimental findings. Figure 3 shows a comparison between these correlations and the results provided by the present numerical model. Particularly, this figure shows the evolution of the radius against time for a spherical bubble of water immersed in a liquid medium 5 °C superheated. As can be seen, this figure shows that the developed numerical based on the level set method model provides reasonably accurate results.

Once it was validated, the numerical model was used to study the governing mechanisms responsible for CO_2_ absorption, such as diffusion, solubility, convection, chemical reactions, and droplet evaporation. The results are outlined below.

## 3. Results

**Figure 4:**
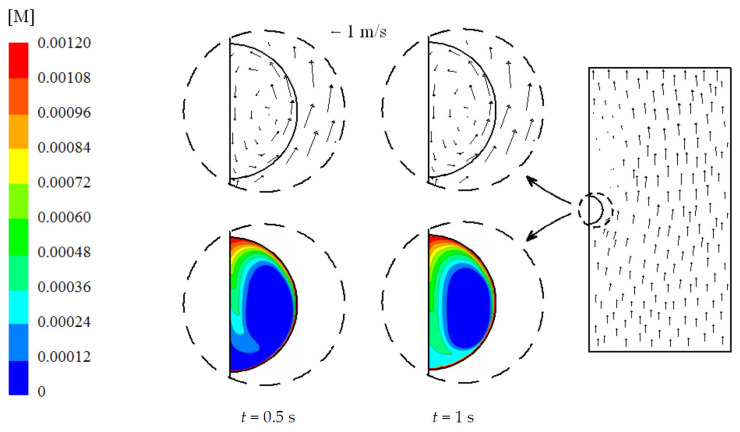
Velocity field and concentration of CO_2_ for time (t) 0.5 and 1 s. 5% initial ammonia concentration, 100 °C initial gas temperature, 5 °C initial liquid temperature, and 1000 μm initial droplet diameter.

**Figure 5:**
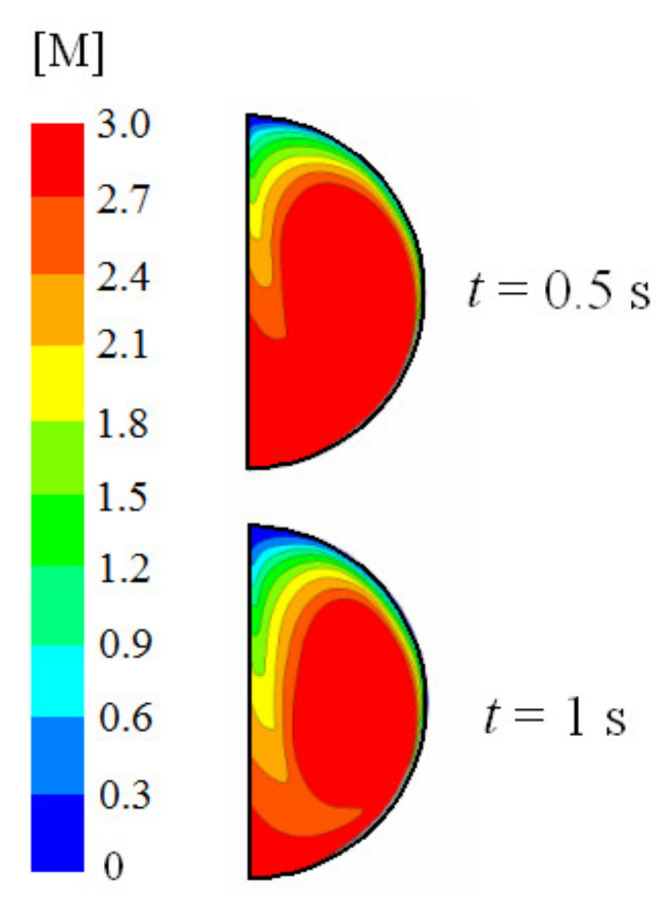
Concentration of NH_3_ for time (t) 0.5 and 1 s. 5% initial ammonia concentration, 100 °C initial gas temperature, 5 °C initial liquid temperature, and 1000 μm initial droplet diameter.

All the results showed in this section will be based on velocities corresponding to usual values observed in practical applications [4,5], 2 m/s initial liquid droplet velocity and 0.5 m/s initial gas velocity. This leads to an initial free stream velocity of 2.5 m/s. An initial 15% *v*/*v* CO_2_ concentration was considered. Figure 4 shows the velocity and concentration of CO_2_ at 0.5 and 1 s using 5% initial ammonia concentration, 100 °C initial gas temperature, 5 °C initial liquid temperature, and 1000 μm initial droplet diameter. The concentration is expressed in M, that is, mol/L. Initially, the CO_2_ concentration inside the droplet is zero and its concentration at the droplet surface obeys Henry’s law. An internal vortex is rapidly induced by the shear stress along the droplet surface and CO_2_ is transported to the core. Radial diffusion and chemical reactions also take place. Figure 4 clearly shows that CO_2_ is transported from the interface to the core of the droplet due to convection. Once CO_2_ is captured into the droplet, it reacts with NH_3_. NH_3_ is consumed first at the interface and then at the core of the droplet, as can be seen in Figure 5. This figure illustrates the NH_3_ concentration at 0.5 and 1 s.

### 3.1. Effect of Convection and Diffusion under Several Initial Droplet Diameters

**Figure 6:**
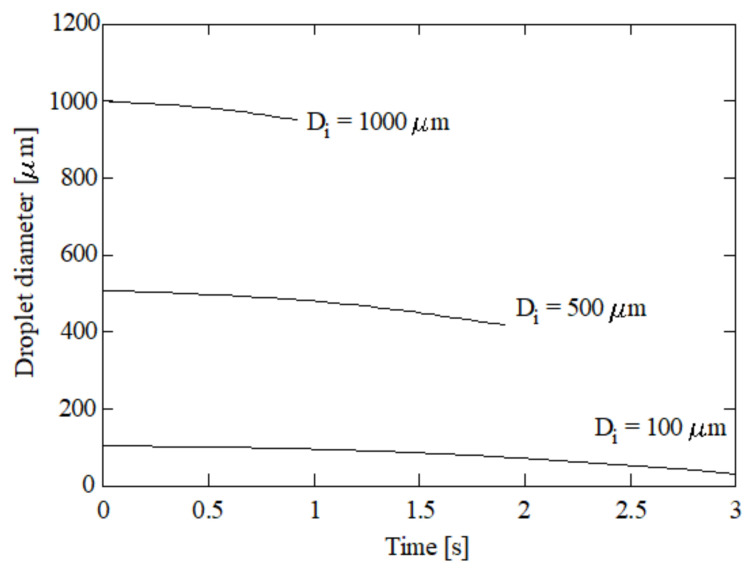
Droplet diameter against time for several initial diameters. 5% initial ammonia concentration, 100 °C initial gas temperature, and 5 °C initial liquid temperature.

Figure 6 shows the droplet diameter against time for 1000, 500, and 100 μm initial diameters. As expected, all droplets experience a reduction in their diameters due to the evaporation phenomena implemented in the model. All droplets remained practically spherical during the entire simulation due to the importance of the surface tension in comparison with the inertial force. Other authors also observed spherical shapes for droplets falling in a gas phase when the diameters are less than 1000 μm approximately [23]. Elperin and Fomykh [37] observed spherical shapes until 1100 μm diameter. Akbar [38] mentioned a 1000 μm limit for limestone droplets, and beyond this value they observed deformations of the droplet and oscillation at the interface. This limit was also mentioned in the numerical study of Amokrane and Caussade [39]. Clift et al. [40] reported that water droplets smaller than 1000 μm falling in air can be considered as rigid spheres.

**Figure 7:**
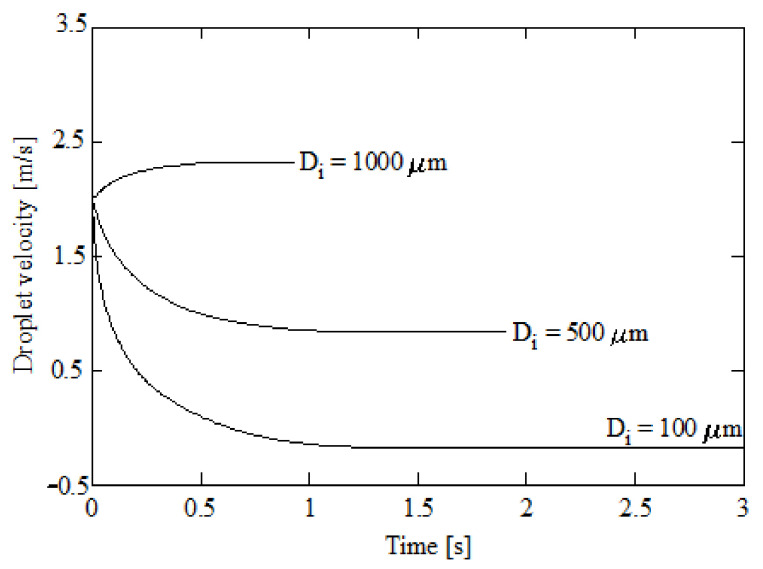
Droplet velocity against time for several initial diameters; 5% initial ammonia concentration, 100 °C initial gas temperature, and 5 °C initial liquid temperature.

**Figure 8:**
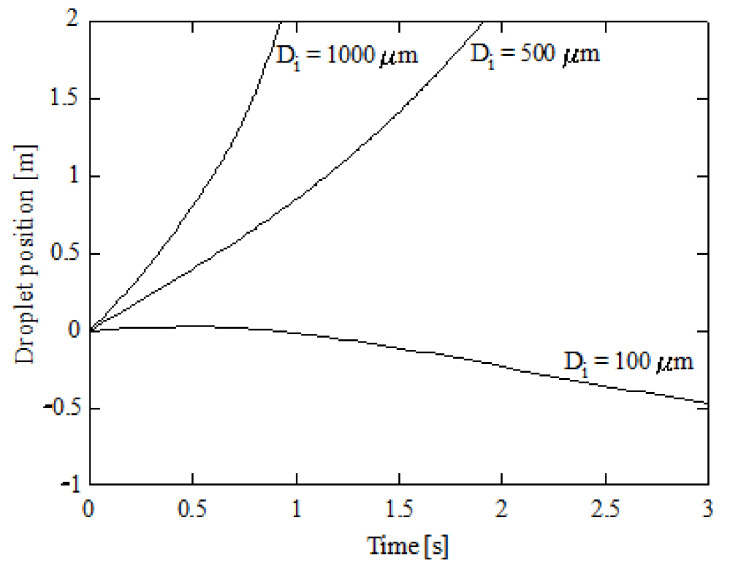
Droplet position against time for several initial diameters; 5% initial ammonia concentration, 100 °C initial gas temperature, and 5 °C initial liquid temperature.

The droplet velocity and position for these initial diameters can be found in Figure 7 and Figure 8 respectively. Figure 6, Figure 7 and Figure 8 correspond to 15% initial CO_2_ concentration, 5% initial ammonia concentration, 100 °C initial gas temperature, and 5 °C initial liquid temperature. As scrubber length, a typical value of 2000 mm [4,5] was assumed. As can be seen in Figure 8, the larger droplet, with an initial diameter of 1000 μm, has a residence time of 0.87 s. This is the time needed to reach 2000 mm in length assumed as height of the scrubber. The droplet with initial diameter of 500 μm needs 1.81 s to reach a 2000 mm length and the droplet with a 100 μm diameter reverses its trajectory. That is, the weight is small enough for the droplet to be displaced upwards instead of downwards. As expected, lower diameters correspond to lower velocities. As shown in Equation (26), two effects are responsible for the droplet trajectory: gravity and drag. Larger droplets are associated with high gravity forces (high weights) and, thus, to high velocities. Nevertheless, as the droplet diameter is reduced, i.e., the weight is reduced, gravity plays a lesser role and the velocity tends to decay. As can be seen in Figure 7, the terminal velocities for the droplets with initial diameters of 1000, 500, and 100 μm are 2.38, 0.81, and −0.19 m/s respectively. The largest droplets, corresponding to 1000 μm, increments its initial velocity due to their important weights, while the other droplets, 500 and 100 μm initial diameters, reduce their initial velocity due to their reduced weight.

**Figure 9:**
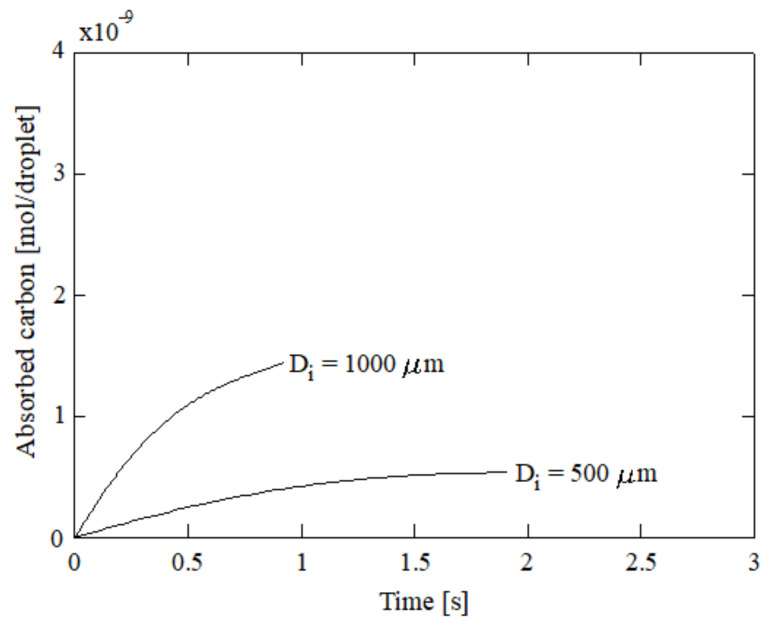
Carbon absorbed by a droplet for several initial diameters; 5% initial ammonia concentration, 100 °C initial gas temperature, and 5 °C initial liquid temperature.

The quantity of carbon absorbed by each droplet is given in Figure 9. Here, the droplet with an initial diameter of 100 μm was discarded due to the inversion of its trajectory. As can be seen, larger droplets are associated with higher carbon absorption even though their residence time is lower. Nevertheless small droplets are associated with lower carbon absorption.

**Figure 10:**
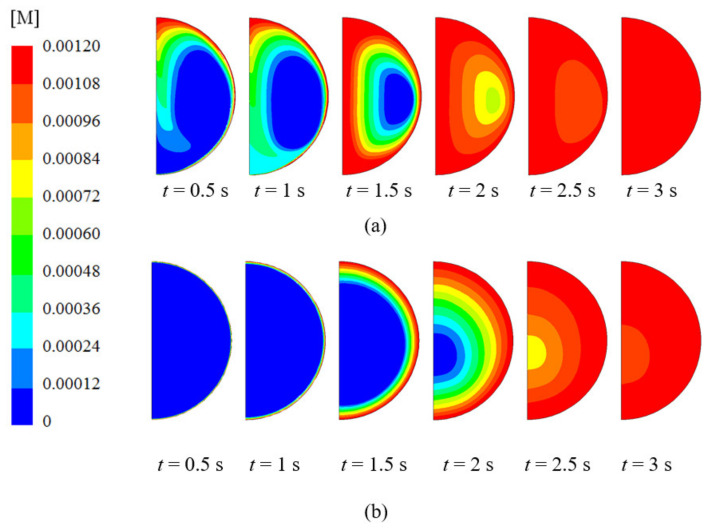
Concentration of CO_2_ against time (t); (**a**) 1000 μm initial diameter; (**b**) 500 μm initial diameter. 5% initial ammonia concentration, 100 °C initial gas temperature, and 5 °C initial liquid temperature.

Figure 10a,b provide the concentration of CO_2_ for droplets with initial diameters of 1000 and 500 μm respectively between 0.5 and 3 s (in case it was possible for the residence time to be 3 s. As mentioned earlier, the droplets with a 1000 μm diameter reach the lower part of the column before 3 s elapse). As can be seen, the droplet with the smaller diameter, 500 μm, presents a distribution of CO_2_ dominated by diffusion since the time evolution of the concentration field is very close to what would be observed in a purely diffusive process: it is almost spherically symmetric. The droplet with the larger initial diameter, 1000 μm, presents a distribution of CO_2_ in a first stage dominated by convection with a highly asymmetric profile. For this droplet, initially CO_2_ is transported by convection in the periphery, depleting the diffusion concentration boundary layer and enhancing mass transfer. After that, it penetrates the droplet due to diffusion.

### 3.2. Effect of Physical and Chemical Absorption

**Figure 11:**
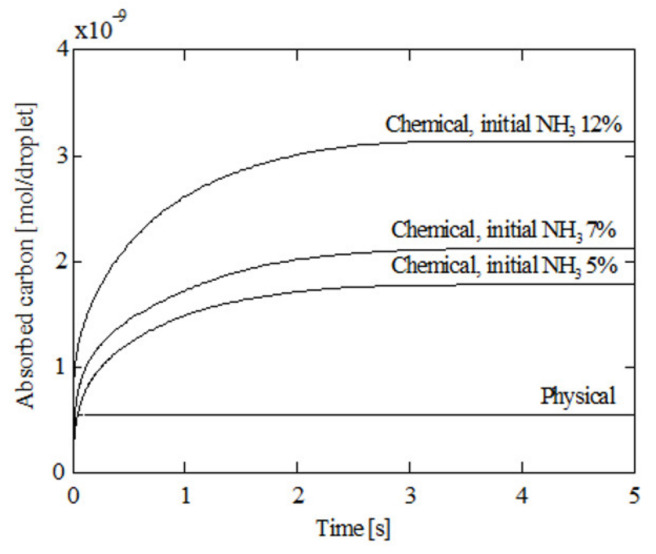
Absorbed carbon under physical and chemical absorption; 5% initial ammonia concentration, 100 °C initial gas temperature, 5 °C initial liquid temperature, and 1000 μm initial diameter.

It is interesting to analyze in detail the effect of chemical dissociation. To this end, Figure 11 compares physical and chemical absorption, that is, in the absence and presence of chemical dissociation, respectively, at a 100 °C initial gas temperature, 5 °C initial liquid temperature, and 1000 μm initial diameter. The absorbed carbon was computed as the sum of moles of CO_2_, NH_2_COOH, and H_2_CO_3_. As can be seen, initially the four curves are extremely similar since CO_2_ is the main species in the droplet. As time progresses, the chemical absorption becomes more intense than the physical one due to the formation of NH_2_COOH and H_2_CO_3_. The absorption of carbon from the flue gas is increased. In the case of 12% initial NH_3_ concentration, an absorption increase of 472.7% over physical absorption was obtained. Another conclusion that can be obtained from Figure 11 is that chemical dissociation prolongs the carbon capture process because more time is needed to reach the saturation state.

### 3.3. Effect of Evaporation and Temperature

**Figure 12:**
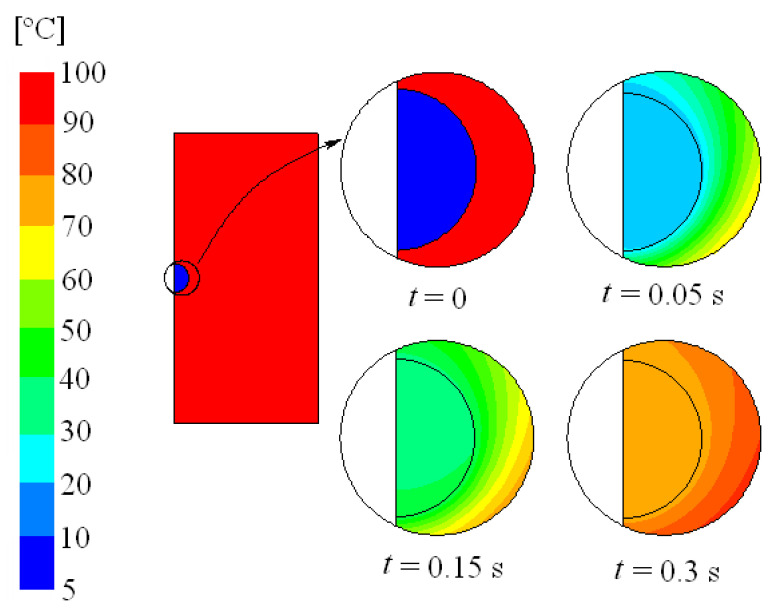
Temperature field for time (t) 0, 0.05, 0.15, and 0.3 s; 5% initial ammonia concentration, 100 °C initial gas temperature, 5 °C initial liquid temperature, and 1000 μm initial diameter.

**Figure 13:**
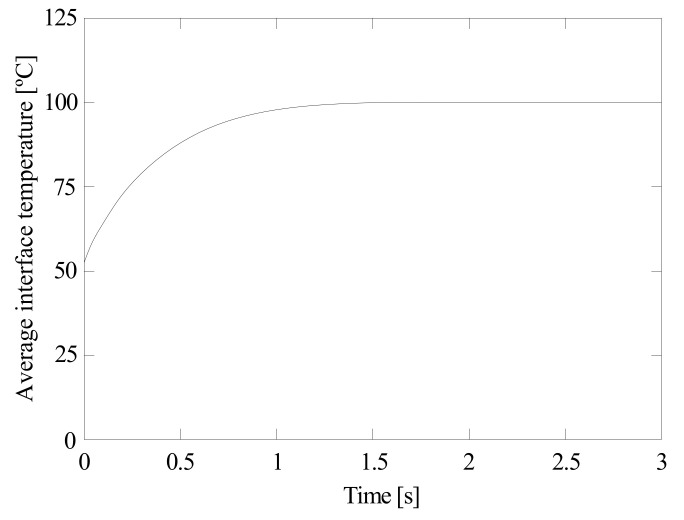
Average interface temperature. 5% initial ammonia concentration, 100 °C initial gas temperature, 5 °C initial liquid temperature, and 1000 μm initial diameter.

It is interesting to analyze the effect of evaporation on the absorption process. Evaporation is closely related to temperature. In practical applications, the liquid can be easily injected at ambient temperature but the gas resulting from combustion remains at a high temperature. An increase in the temperature facilitates the forward reactions of Equations (3)–(5) and, thus, NH_4_HCO_3_ and H_2_CO_3_ formation, leading to improve carbon absorption. Nevertheless, a rise in temperature increases the interface temperature and the Henry’s constant is reduced. This lowers the amount of CO_2_ at the interface and, with it, the absorption process. Figure 12 shows the liquid and gas temperature against time at 5% initial ammonia concentration, 100 °C initial gas temperature, 5 °C initial liquid temperature, and 1000 μm initial diameter. As can be seen, the liquid is heated and the gas is cooled once they are in contact. The variation in the liquid temperature is more pronounced than that in the gas temperature since water has a higher heat capacity than gas. The average interface temperature against time is represented in Figure 13 (in case it was possible for the residence time to be 3 s).

**Figure 14:**
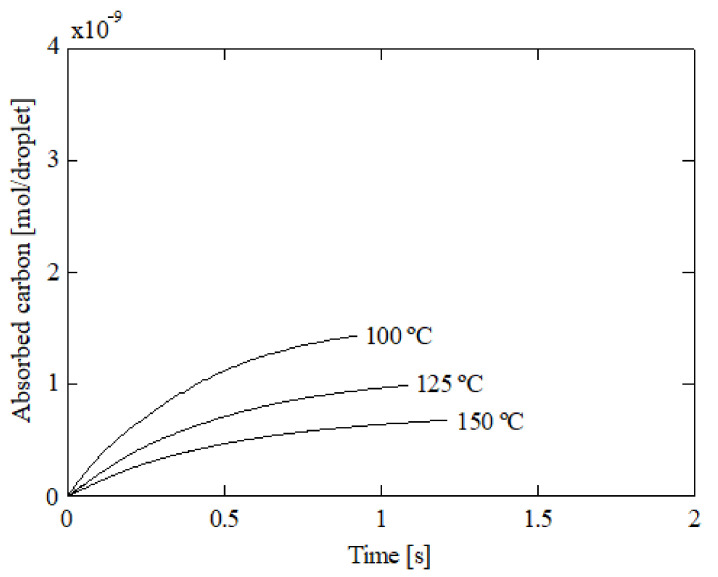
Absorbed carbon by a droplet for several initial gas temperatures; 5% initial ammonia concentration, 5 °C initial liquid temperature, and 1000 μm initial diameter.

**Figure 15:**
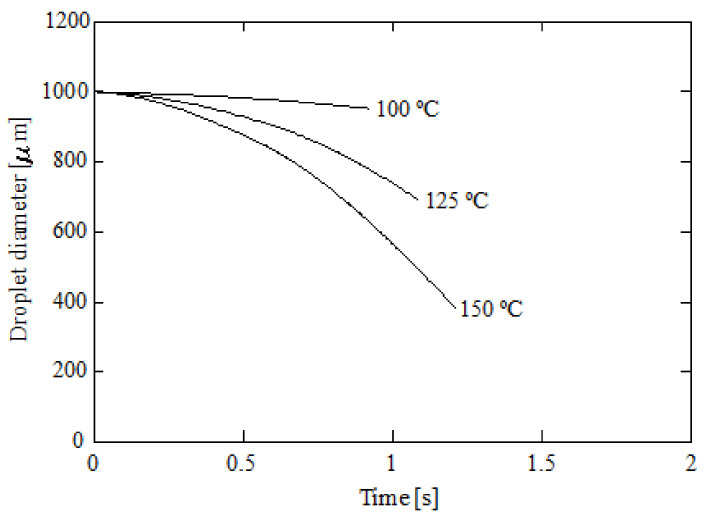
Droplet diameter against time for several initial gas temperatures; 5% initial ammonia concentration, 5 °C initial liquid temperature, and 1000 μm initial diameter.

Figure 14 shows the carbon absorbed by a droplet against time at a 5% initial ammonia concentration, 5 °C initial liquid temperature, and 1000 μm initial diameter. Three gas temperatures were represented: 100, 125, and 150 °C. As can be seen, the carbon absorption decreases with the gas temperature due to a reduction in the Henry’s constant. Previous works also revealed similar trends using ammonia [8] and other solvents such as DEA and AEEA [41]. Another effect that decreases carbon absorption is evaporation. If the gas temperature is increased, the droplet’s diameter is reduced due to evaporation. Figure 14 shows the absorbed carbon for 150 °C than 100 °C. As can be seen, higher temperatures promote higher residence times due to lower velocities, but the droplet diameter is reduced and, thus, the CO_2_ absorption. In Figure 15, the droplet diameter against time is provided for 100, 125, and 150 °C. As can be seen, the droplet diameter experiences a considerable reduction with the gas temperature. The main consequence is a reduction in the absorbed carbon. Particularly, a 48.7% reduction in the absorbed carbon was obtained from 100 to 150 °C. The simulations were repeated using a constant diameter and the reduction in carbon absorption was 41.9%, which confirms the role played by evaporation.

**Figure 16:**
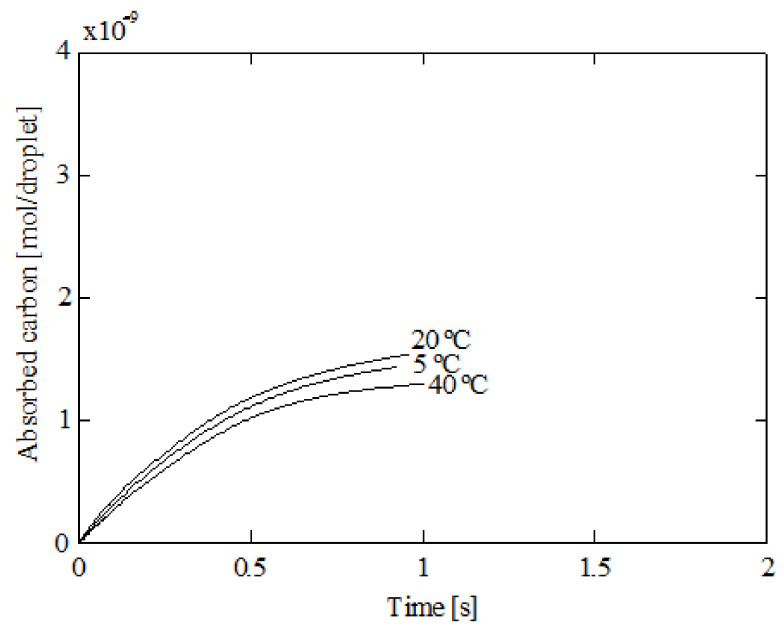
Absorbed carbon by a droplet for several initial liquid temperatures; 5% initial ammonia concentration, 100 °C initial gas temperature, and 1000 μm initial diameter.

The influence of the liquid temperature is illustrated in Figure 16, which shows the carbon absorption corresponding to a 5, 20, and 40 °C liquid temperature. In this figure, it can be seen that the carbon absorption is higher for 20 °C and lower for 40 °C.

## 4. Conclusions

This work presents a numerical analysis to study CO_2_ capture by ammonia. Aqueous ammonia was employed as absorbent due to its high absorption capacity, low energy requirement for regeneration, and good inoxidability. The results obtained can be channeled to the improvement of equipment operation and efficiency such as absorption and stripper columns, as well as the process of regeneration of absorbents.

A single droplet was studied. The analysis of the time evolution of the species, velocity and temperature provided information about the governing psychochemical phenomena, such as diffusion, solubility, convection, chemical reactions, and evaporation. It was found that diffusion prevails over convection, especially for small droplets. Convection is important in the initial instants of the process. Chemical reactions increment the absorption rate up to 472.7% in comparison with physical absorption alone for the parameters analyzed in the present work. Chemical reactions enhance the mass transfer and delay the saturation state. For the parameters studied, it was found that the droplet evaporation reduces the absorption rate by up to 41.9%. The flue gas temperature has a considerable influence on both evaporation and absorption.

It is worth noting that the main drawback of the present work is that it neglects ammonia evaporation, since this will evaporate much faster than water and, thus, its concentration is affected. Ammonia might escape from the solution before the reaction with CO_2_, and this phenomenon will be object of analysis in future works. Another areas for future research work will be analyzing larger droplets and the effect of the exhaust gas humidity.

## Figures and Tables

**Table 1:** Forward and backward constants.

Reaction	Forward Rate Constant	Backward Rate Constant
Equation (3)	$\ln(k_{f1})=25.6278-\frac{44.988}{RT}$	$\ln(k_{b1})=0.0078-\frac{5.3602}{RT}$
Equation (4)	$\ln(k_{f2})=2.4078-\frac{15.851}{RT}$	$\ln(k_{b2})=4.6425-\frac{27.624}{RT}$
Equation (5)	$\ln(k_{f3})=e^{22.6-7799/T}-\ln(C_{H_{2}O})$	$\ln(k_{b3})=30.15-8018/T$

## Data Availability

Not applicable.
